# Dynamics and evolution of the inverted repeat-large single copy junctions in the chloroplast genomes of monocots

**DOI:** 10.1186/1471-2148-8-36

**Published:** 2008-01-31

**Authors:** Rui-Jiang Wang, Chiao-Lei Cheng, Ching-Chun Chang, Chun-Lin Wu, Tian-Mu Su, Shu-Miaw Chaw

**Affiliations:** 1 South China Botanical Garden, the Chinese Academy of Sciences, Guangzhou 510650, China; 2 Research Center for Biodiversity, Academia Sinica, Taipei 115, Taiwan; 3 Institute of Biotechnology, National Cheng Kung University, Tainan, Taiwan

## Abstract

**Background:**

Various expansions or contractions of inverted repeats (IRs) in chloroplast genomes led to fluxes in the IR-LSC (large single copy) junctions. Previous studies revealed that some monocot IRs contain a *trnH-rps19 *gene cluster, and it has been speculated that this may be an evidence of a duplication event prior to the divergence of monocot lineages. Therefore, we compared the organizations of genes flanking two IR-LSC junctions in 123 angiosperm representatives to uncover the evolutionary dynamics of IR-LSC junctions in basal angiosperms and monocots.

**Results:**

The organizations of genes flanking IR-LSC junctions in angiosperms can be classified into three types. Generally each IR of monocots contains a *trnH-rps19 *gene cluster near the IR-LSC junctions, which differs from those in non-monocot angiosperms. Moreover, IRs expanded more progressively in monocots than in non-monocot angiosperms. IR-LSC junctions commonly occurred at polyA tract or A-rich regions in angiosperms. Our RT-PCR assays indicate that in monocot IR_A _the *trnH-rps19 *gene cluster is regulated by two opposing promoters, *S10*_*A *_and *psbA*.

**Conclusion:**

Two hypotheses are proposed to account for the evolution of IR expansions in monocots. Based on our observations, the inclusion of a *trnH-rps19 *cluster in majority of monocot IRs could be reasonably explained by the hypothesis that a DSB event first occurred at IR_B _and led to the expansion of IRs to *trnH*, followed by a successive DSB event within IR_A _and lead to the expansion of IRs to *rps19 *or to *rpl22 *so far. This implies that the duplication of *trnH-rps19 *gene cluster was prior to the diversification of extant monocot lineages. The duplicated *trnH *genes in the IR_B _of most monocots and non-monocot angiosperms have distinct fates, which are likely regulated by different expression levels of *S10*_*A *_and *S10*_*B *_promoters. Further study is needed to unravel the evolutionary significance of IR expansion in more recently diverged monocots.

## Background

Typically the cpDNAs of land plants contain two identical segments, the inverted repeats (IRs: IR_A _and IR_B_), separated by two single copy (SC) sequences, the large single copy (LSC) region and the small single copy (SSC) region [1,2]. Thus four junctions, termed J_LA_, J_SA_, J_SB_, J_LB_, are between the two IRs and the SC regions [3,4]. A major constraint on cpDNA is its organization into large clusters of polycistronically transcribed genes [5-7]. As a result, large structural changes in cpDNA, such as segmental duplication or deletion and mutation in gene order, are relatively rare and evolutionarily useful in making phylogenetic inferences [8].

In land plants, the sizes of rRNA gene-containing IRs are notably variable, ranging from 10 kb in liverworts to 20–25 kb in most angiosperms [2,9,10], and up to 76 kb in *Pelargonium *(a eudicot) [11]. Successive IR expansions, either within angiosperms or between non-vascular plants and angiosperms, have led to floating of J_LA _and J_LB _[12] and have evolutionary significance [13-15]. Several models concerning the expansion and contraction of IR regions have been proposed to explain the possible mechanisms that result in shift of the IR-LSC junctions. For example, the unusual triple-sized expansion of the *Geranium *IR was hypothesized as an outcome of inversion due to recombination between homologous dispersed repeats [16]. Similarly, the at least 4 kb expansion of the IR in buckwheat (*Fagopyrum esculentum*) cpDNA was also considered to be associated with an inversion [17].

Goulding et al. [15] found that in most *Nicotiana *species IR regions have both expanded and contracted with slight variations in length during the evolution of the genus. The exception is *N. acuminata*, which underwent a large IR expansion of over 12 kb. Goulding et al. [15] proposed two mechanisms of IR expansion: (i) gene conversion to account for the small IR expansion or movements in most species of the genus, and (ii) a DNA double-strand break (DSB) to explain the extensive incorporation of the LSC region into the IR of *N. acuminata*. Perry et al. [18] analyzed the endpoint sequence of a large 78 kb rearrangement in adzuki bean (*Vigna angularis*) and concluded that the unusual organization was caused by a two-step process of expansion and contraction of the IR, rather than a large inversion.

Recent phylogenetic studies using various molecular markers have yielded robust support for the hypothesis of either *Amborella *alone or *Amborella-*Nymphaeales together as the basal-most clade of angiosperms [13,19-26], and the genus *Acorus *has been identified as the earliest splitting lineage in monocots. However, the sister group of monocots is still unclear [26].

Monocots include about one-fourth of the world's flowering plants and represent one of the oldest angiosperm lineages [27]. However, no comparative study has been conducted to investigate the diversity and evolutionary dynamics at the IR-LSC junctions of cpDNAs in basal angiosperms and monocots as a whole. Goulding et al. [15] found that each IR in rice and maize (Poaceae) contains a fully duplicated *trnH-rps19 *gene cluster. Chang et al. [20] further discovered that the IRs of two other remote monocot taxa, *Acorus *and Orchidaceae, also include *trnH *and *rps19 *(although the 3*' *region of *rps19 *was truncated in *Acorus*), and speculated that the clustering of *rps19 *and *trnH *was probably duplicated before the diversification of extant monocot lineages.

**Figure 1:**
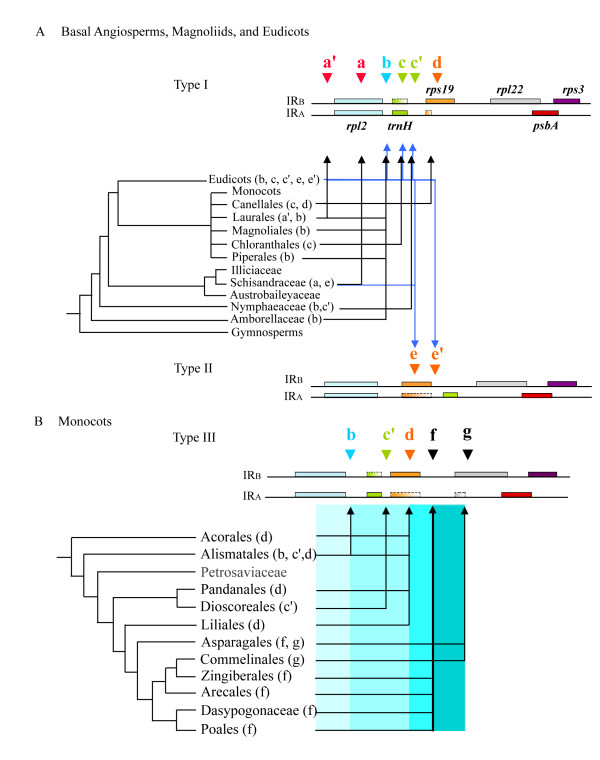
Classification of IR-LSC junction types based on the organization of genes flanking J_LB_ and J_LA_ in angiosperms. Triangles coded by different colors and letters indicate various locations of IR-LSC junctions in corresponding angiosperm lineages. Shaded boxes denote protein-coding genes, and boxes with broken margins and gradient color stand for genes that are variable in length. Relationships of major non-monocot (A) and monocot (B) lineages followed the phylogenetic trees of Soltis et al. (2005) [27]. (A) In type I the IR-LSC junction is located downstream of *rpl2 *and upstream of *rps19 *. In type II *rps19 *is located downstream of *rpl2 *in IR_A_. (B) In type III each IR has a copy of the *trnH-rps19 *cluster, although in the IR_A_ regions the *rps19 *genes are variously truncated at the 3*' *regions in sampled taxa. The blue gradient on the right side of the monocot phylogenetic tree denotes the progressively expanded IRs.

As a result of expansion and contraction, the IRs in the cpDNA of angiosperms have been suggested as an evolutionary marker for elucidating relationships among some taxa [14,28]. To improve understanding of the dynamics and evolution of IR-LSC junctions from basal angiosperms to the emergence and diversification of monocots (assuming that this evolutionary course is correct), we sampled 52 key species and determined the sequences of the two regions spanning J_LA _(Fig. 1, between the 3*' *end of *rpl2 *and the 5*' *end of *psb*A) and J_LB _(Fig. 1, between the 3*' *end of *rpl2 *and the 5*' *end of *rpl22*). A total of 123 representative angiosperms, including 12 basal angiosperms, 16 magnoliids, 62 eudicots, and 33 monocots (see the additional file 1), were analyzed. Three types of gene arrangements flanking the J_LA _and J_LB _regions were recognized and mapped onto the angiosperm phylogeny. In order to explain this arrangements we propose two alternative hypotheses concerning the evolutionary history of the flux of IR-LSC. Furthermore, to verify the transcriptional status of the duplicated *trnH-rps19 *gene cluster near the IR_A _junctions the activity of two operons in *Asparagus densiflorus*, *S10*_*A *_and *psbA*, was investigated.

## Results

Several terms used in this section are briefly explained here. Types of IR-LSC junction are based on the organization of genes flanking J_LB_ and J_LA_ in angiosperms. Type I is found in most non-angiosperm dicots. It refers to an intact *trnH *gene being located directly downstream of the *rpl2 *sequence in IR_A _and an intact *rps19 *gene being located directly downstream of the *rpl2 *sequence in IR_B_. No full-length *rps19 *or *trnH *sequence is present in IR_A _or IR_B _respectively. Type II refers to a partial sequence of *rps19 *being located directly between *rpl2 *and *trnH *in IR_A_. Type II pattern is only found in some eudicots while type III characterizes the IRs of most monocots, in which each IR contains a *trnH-rps19 *cluster. The letters a, a', c, ... and g used in the text and in Figure 1 refer to the IR-LSC junctions found in cpDNAs of sampled angiosperms.

### In non-monocot angiosperms IR-LSC junctions of IR_B _are largely located between *rpl2 *and *rps19*

Figure 1 shows that the IR-LSC junctions in 90 non-monocot angiosperms usually drift around position b (data shown in the additional file 1). In these cases, designated as type I, an intact *trnH *gene is always present near the J_LA _but absent from the J_LB_. In *Chloranthus oldhami*, *C. spicatus*, *Sarcandra glabra *(Chloranthales), *Canella winterana *(Canellales), *Ranunculus japonica *and *R. macranthus *(eudicot), a partial *trnH *sequence is found extending to position c in IR_B _(Fig. 1A, additional file 1). The IR-LSC junctions were located upstream of position c' (i.e. upstream of *trnH*) in *Nuphar advena *(Nymphaeaceae) and *Elaeagnus formosana *(Elaeagnaceae, eudicot), at position a in *Kadsura japonica *(Schisandraceae, Austrobaileyales), and at position a' in *Calycanthus fertilis *and *C. floridus *(Calycanthaceae, Laurales, [29,30]) (Fig. 1A). However, *Vitis vinifera *(Vitaceae, eudicot) showed a complete loss of *rpl2 *near J_LA _[31].

The Winteraceae (Canellales), exemplified by *Zygogynum pauciflorum *and *Drimys granadensis *[29], were exceptional in that the organization of the genes flanking the IR-LSC junctions resembled the one found in most monocots, rather than the organization seen in other non-monocot angiosperms. Notably, each of their IRs contained a *trnH-rps19 *cluster and their IR-LSC junctions were located within the 5*' *portion of *rps19 *(position d, Fig. 1).

Type II IR-LSC junctions were found in *Schisandra arisanensis *(Schisandraceae; Austrobaileyales) and some 41 representative eudicots (Fig. 1A; additional file 1). Unlike type I, the J_LA _of type II shifted to the 5*' *end of the truncated *rps19 *in IR_A _(position e and e', Fig. 1A, additional file 1).

### IRs of monocots generally contain *trnH-rps19 *clusters

In contrast to basal angiosperms and eudicots, most monocots (Fig. 1B) had *trnH-rps19 *clusters present in each of the two IRs, and the IR-LSC junctions were generally at position f (Arecales, Dasypogonaceae, *Asparagus densiflorus *[Liliales], Poales and Zingiberales) or g (in Asparagales and Commelinales) (Fig. 1B). This type of gene organization was classified as type III. In addition, IR-LSC junctions of some monocots were located downstream of *rpl2 *(position b; in Araceae, most Alismataceae, and Hydrocharitaceae), of *trnH *(position c' in Potamogetonaceae and Dioscoreaceae), or within *rps19 *(position d, Fig. 1; in Acorales, *Lilium formosamum *[Liliales] and Panadanales). When the IR-LSC junction was at position d, the *rps19 *sequence in IR_A _was found to be partially truncated most of the times.

### Sequences flanking IR-LSC junctions are more variable in monocots than in non-monocot angiosperms

**Figure 2:**
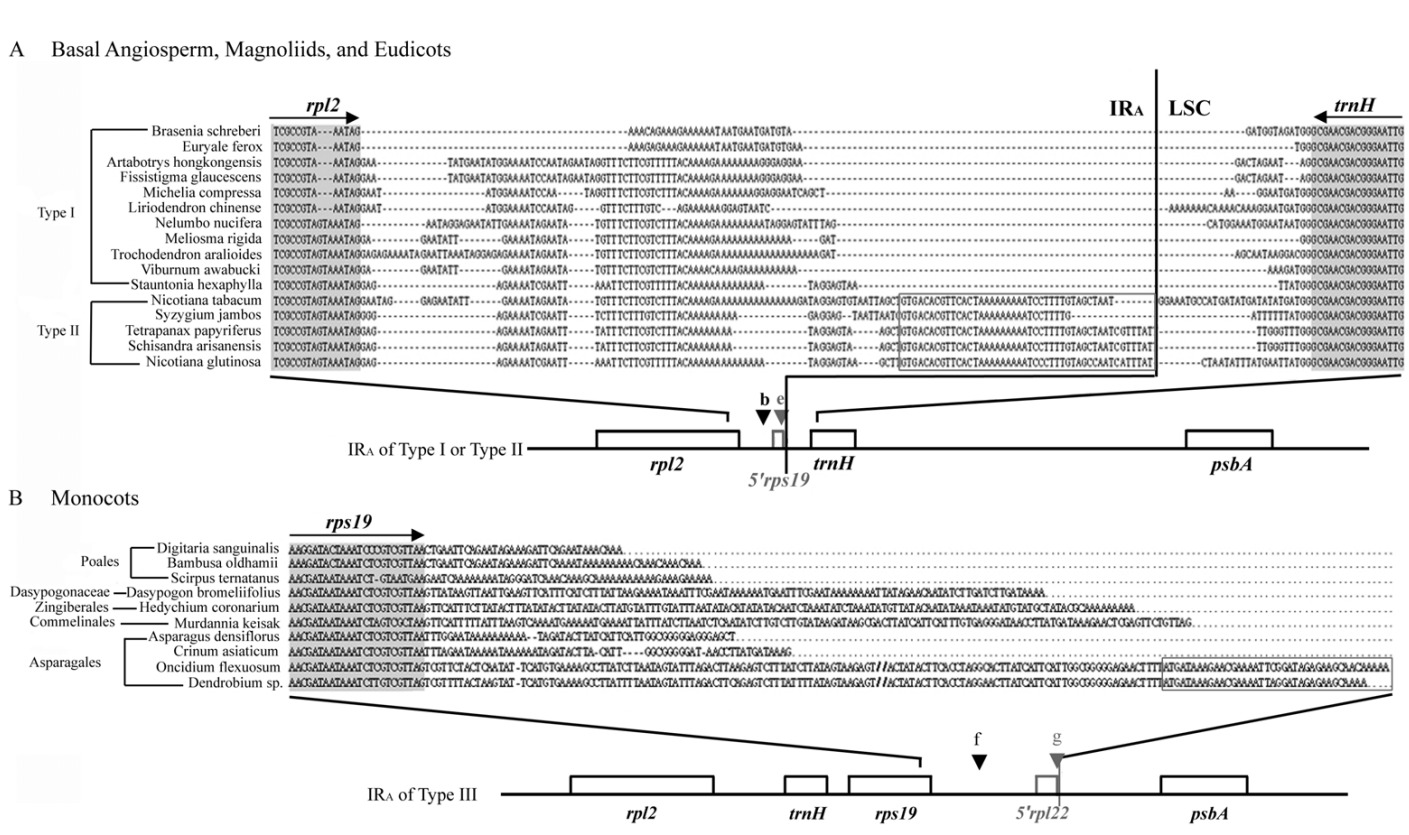
Alignment of sequences flanking J_LA_ regions in some basal angiosperms, Magnoliids, and eudicots (type I at position b, and type II at position e), and the sequences within the J_LA _in some monocots (type III at position f or g). Dashed lines denote gaps. Grey segments and the arrow lines above indicate coding regions and transcriptional directions of specified genes, respectively. (A) Grey box denotes degenerate *rps19 *genes (5*' *segment) found in the IR_A_ of the type II (position e) pattern. (B) A degenerate *rpl22 *gene (boxed sequences) found in the IR_A _of type III (position g). "//" stands for abbreviated base pairs in the sequences of *Oncidium *and *Dendrobium*.

Figure 2 illustrates alignment of the sequences flanking the J_LA _regions in some representatives of basal angiosperms and eudicots (A) and monocots (B). Of particular interest is the observation that the IR-LSC junctions of basal angiosperms, eudicots and monocots are commonly found at either polyA tract or A-rich regions (Fig. 2). We also found that the dicot IR sequences near the IR-LSC junctions varied little and could be aligned among orders having the same or different IR-LSC junction types, while in monocots the corresponding regions were very different and difficult to align across different orders (Fig. 2B). Moreover, within the sampled angiosperm families the sequences flanking the J_LA_regions were very similar.

### Transcription of monocot *trnH-rps19 *of IR_A _is regulated by both chloroplast *S10*_*A *_and *psbA *promoters

Among the chloroplast operons, the *S10 *ribosomal protein operon is the largest. It contains genes encoding both small (*rps*) and large (*rpl*) ribosomal protein subunits that are organized into a polycistronic transcription unit conserved in known cpDNAs [32]. In angiosperms, the 5*' *end of the *S10 *operon is initiated within the IR, but only in IR_B _does the operon extend into the LSC region, and the *S10 *operon is only partially in IR_A _(*viz*. the *S10*_*A *_operon). However, a second operon in IR_A_, the *psbA *operon, is transcribed from LSC towards IR_A _[32] and opposite to the *S10*_*A *_operon.

**Figure 3:**
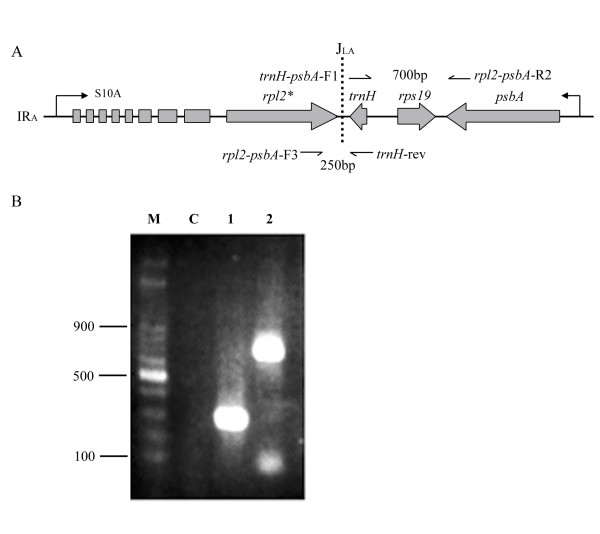
Transcription analysis of the *S10 *and *psbA *operons in a monocot representative, *Asparagus densiflorus*. (A) The relative position of the *S10 *and *psbA *operons at the flanking region of the IR_A_-LSC junction. An arrow line denotes the transcriptional direction. One-side arrow lines indicate primers. (B) Transcripts obtained by reverse transcription PCR (RT-PCR). Lane M, 100 bp ladder; lane C, negative control using the same RNA as the template in lanes 1 and 2; lane 1, RT-PCR with the primer pair *trnH*-rev and *rpl2-psbA-*F3; lane 2, RT-PCR with the primer pair *trnH-psbA*-F1 and *rpl2-psbA-*R2.

In the Winteraceae and a majority of monocots, the *trnH-rps19 *cluster of IR_A _is included in both the *S10 *and *psbA *operons. Therefore, this gene cluster may be regulated by two opposing promoters, the *S10*_*A *_and the *psbA *(Fig. 3A). In monocots, if the *trnH *in IR_A _is indeed regulated by the above-mentioned two opposing promoters, the function of the *trnH *gene may be repressed because antisense-*trnH *RNAs would be generated by both the *S10*_*A *_and *S10*_*B *_promoters. To verify this possibility, we conducted RT-PCR assays using specific primers for a type III representative, *Asparagus densiflorus*, with the IR-LSC junction located at position f (Fig. 1B).

Our results indicate that expression of the *trnH *gene in IR_A _is regulated by both the *S10*_*A *_and *psbA *promoters. This suggests that the duplicated *trnH *gene located in the IR_B _region of most monocots and in some non-monocots has different fates (i.e. functional or degenerate in different lineages; see Fig. 1). Figure 3B shows that two RT-PCR products, a 250 bp and a 700 bp fragment, respectively, were generated when specific primer pairs for each were used (Fig. 3A). The former fragment was amplified from the transcripts made by the *psbA *promoter, and the latter by the *S10 *promoter. This result confirms that the *trnH-rps19 *cluster of IR_A _is regulated by two opposing promoters (Fig. 3B), indicating that the transcription machinery in IRs of monocots may differ from that of basal angiosperms and eudicots.

## Discussion

### Two evolutionary hypotheses for the flux of IR-LSC junctions in monocots

As shown in Figure 1A, IR-LSC junctions of the *Amborella *+ Nymphaeales are mainly located at position b, but junctions of monocots are further expanded to encompass LSC genes and are located at positions f or g. Since the two IRs of monocots usually include the *trnH-rps19 *cluster (position f or g, further downstream of *rpl2*; Fig. 1B), we hypothesize that at least two duplication events are required to explain the expansion of IRs in monocots during the course of IR evolution from an *Amborella*-like ancestor to present-day monocots. If this hypothesis is correct, it is expected that an intermediate junction type could be traceable in the cpDNAs of some early divergent monocot lineages between the two duplication events.

**Figure 4:**
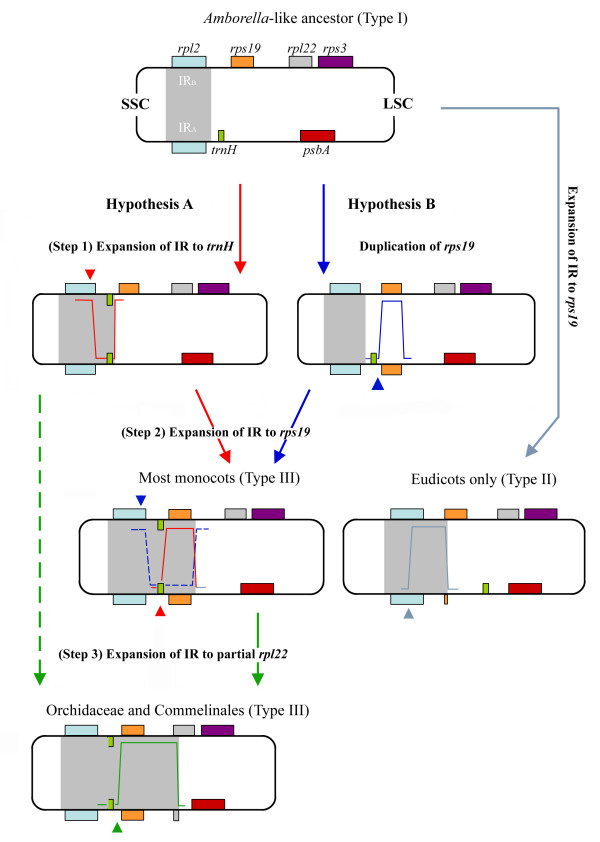
Two hypotheses for the evolutionary derivation of the *trnH-rps19 *cluster in IRs of monocots from an *Amborella-*like ancestor. Arrow lines coded by different colors indicate distinct evolutionary pathways. Arrowheads denote possible breakpoints when DSB events occurred (different DSB colors are associated with different IR expansions). The light blue arrow line refers to a scenario in which a type II IR-LSC junction was established (see Fig. 1) in some eudicots (note that the *rps19 *residue is situated between *rpl2 *and *trnH *in IR_A_). The grey area in each cpDNA molecule highlights the IRs at all evolutionary stages.

Narayanan et al. [33] have recently presented a model of gene amplification in eukaryotes that argues strongly for the involvement of hairpin-capped DSBs in the initiation. Based on this model and our observations, we propose two hypotheses to account for the evolution of IR expansions in monocots (Fig. 4). In hypothesis A, a DSB event (Fig. 4, red arrowhead in step 1) occurs first within the IR_B _of an *Amborella*-like ancestor, and then the free 3*' *end of the broken strand is repaired against the homologous sequence in IR_A_. The repaired sequence extends over the original IR-LSC junction and reaches the area downstream of *trnH *(Fig. 4, step 1), so that duplication of a *trnH *gene in the newly repaired IR_B _is achieved. Similarly, a second DSB event occurs in IR_A _adjacent to the IR_A_-LSC junction (Fig. 4, red arrowhead at step 2) so that duplication of *rps19 *at IR_A _can be initiated, and a *trnH-rps19 *cluster nearby J_LB _(Fig. 4, step 2) is created. The newly formed IRs might cover the *trnH-rps19 *cluster and extend further into the intergenic spacer between *rps19 *and *rpl22 *(Fig. 4, step 1 to step 2). Furthermore, if one additional DSB event took place within the intergenic spacer located between *rps19 *and *rpl22 *in the LSC region, a partial *rpl22 *gene would be duplicated at IR_A _using the *rpl22 *sequence of LSC as a template, and from then on the repaired IRs might have expanded towards the 5*' *region of the *rpl22 *(Fig. 4, step 2 to step 3). The exceptionally long IRs observed in the Orchidaceae and Commelinales are likely to have been generated by this process. The same outcomes could also result if the process proceeded directly from step 1 to step 3 without step 2 (Fig. 4, path indicated by green dashed arrow).

Hypothesis B, on the other hand, assumes that *rps19 *would be duplicated or converted prior to the duplication of *trnH *through a DSB event that takes place at IR_A _first (Fig. 4; blue arrowhead of step 1). A second DSB event (Fig. 4; blue arrowhead of step 2) then would take place within the IR_B _region through a similar repair process to the one mentioned before, so that a duplicated *trnH *is generated at IR_B_. Finally, the IRs expand downstream of *rps19*. In hypothesis B subsequent extension of IRs is assumed to resemble step 3 of hypothesis A.

Duplication of a partial or complete *rps19 *gene was also observed in some eudicots and Schisandraceae (type II) with their respective IR-LSC junctions located at position e or e' (additional file 1; Fig. 1). However, these duplicated *rps19 *genes (both partial and complete) are situated between the *rpl2 *and *trnH *genes of the IR_A _(refer to type II in Fig. 1A and Fig. 4 [see the light blue line at the right side leading to eudicots]) rather than downstream of *trnH *or upstream of *psbA *(refer to step (2) and (3) of hypothesis A in Figure 4). Therefore, the gene arrangement flanking the IR_A_-LSC of type II deviates from that of type I, suggesting that duplication of *rps19 *genes in type II must have a distinct evolutionary history.

**Figure 5:**
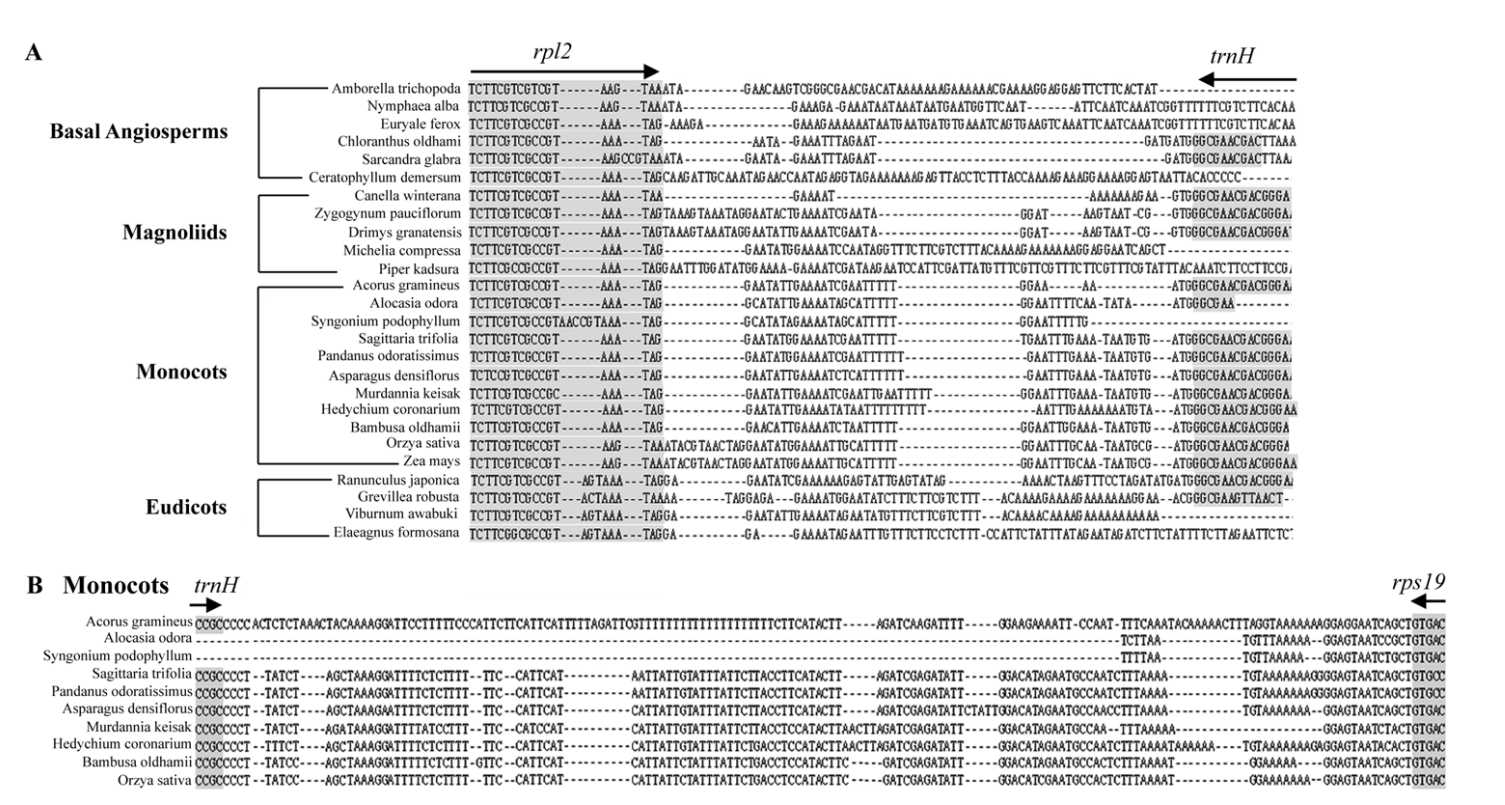
Comparisons of sequences that flank J_LA _regions in angiosperms. (A) Alignment of *rpl2-trnH *intergenic spacers in representative basal angiosperms, magnoliids, monocots, and eudicots. Grey regions and the arrow lines above indicate locations and transcriptional directions of *rpl2 *and *trnH*, respectively. (B) Alignment of the *trnH-rps19 *intergenic spacer sequences at IR_A _strand among representatives of major monocot orders. Grey regions with arrow lines indicate locations and transcriptional directions of *trnH *and *rps19*, respectively.

Based on comparisons of aligned *rpl2-trnH *and *trnH-rps19 *intergenic spacer sequences from representatives of major monocot orders (Figure 5A, B), it is apparent that these two spacer sequences are separately highly similar across the sampled monocot orders. These data give strong support to hypothesis A that in monocots expansion and inclusion of *trnH-rps19 *gene cluster in IRs might require at least two common DSBs (please refer to steps 1 to 3 of hypothesis A in Figure 4): one occurring within IR_B _(refer to Fig. 4, step 1), and the within IR_A _(refer to Fig, 4 step 2 or 3).

However, we did not discover any inverted repeats that might have led to the formation of hairpins in the monocot intergenic spacers of *trnH *and *rps19*. Therefore, we are inclined to conclude that the expansions of monocot IRs took the path depicted in hypothesis A.

### IR expansion may be initiated by DSB and end in the nearby polyA region in angiosperms

Goulding et al. [15] proposed two models to account for two kinds of IR expansion: (1) small and random IR expansions, caused by gene conversion (*viz*. single strand break); and (2) large IR expansions, like those found in the *Nicotiana *species, rice and maize, generated via DSB events. Narayanan et al. [33] further demonstrated that DSBs can trigger gene amplification through a variety of mechanisms, and that breakage at the inverted repeats of chromosomes can cause gene amplification.

After a critical comparison of genes or sequences adjacent to the IR-LSC junctions in 33 major orders and 8 families of angiosperms (following the classification system proposed by Soltis et al. 2005 [27]), we hypothesize that IR expansions resulted principally from the DSB events that occurred during IR evolution from the *Amborella*-like ancestor to monocots. This hypothesis is founded on the following 5 observations: (1) the length of IR expansion from basal angiosperms to monocots is large (more than 100 bp); (2) *trnH *and *rps19 *are situated downstream of IR_A _and IR_B_, respectively, in all sampled basal angiosperms (Fig. 1A). This type of gene arrangement might represent the ancestral gene pattern in basal angiosperms; (3) IRs of several basal angiosperms (e.g. Schisandraceae, Chloranthales and Magnoliales, Winteraceae) and eudicots (Fig. 1A) have partially or completely duplicated *trnH *genes located at IR_B_; (4) in comparison with other angiosperms, monocot IRs have expanded further to include a duplicated *rps19 *in IR_A_, and this expansion should have occurred before the diversification of major monocot orders; and (5) the IRs of advanced monocots (from Asparagales to Poales) have expanded to encompass more LSC sequences or genes (Fig. 1B). Nevertheless, the latter expansions did not apparently result from another common DSB event but from independent ones, because among sampled monocot orders the downstream regions of *rps19 *genes have low sequence similarity (Fig. 2). At the infra-order level of angiosperms, gene conversion might occur frequently at meiosis and cause small IR expansion or contraction during evolution, as found in Apiaceae [14] and *Nicotiana *[15].

Studies on the IR-LSC junctions of *Nicotiana *species [15] and Apiaceous plants [14] have indicated that short repeats or "polyA tract" sequences associated with tRNAs at the IR-LSC boundaries might be likely hotspots for recombination. We also observed that polyA tract sequences are commonly present near the IR-LSC junctions in all the basal angiosperms, eudicots and monocots examined (Fig. 2), indicating that such sequences are closely linked with the dynamics of IR-LSC junctions and expansion of IRs. In this regard, we further propose that IR expansion may initiate at the DSBs and finish at the polyA tract regions, where recombination may actively occur, and that the recombination mechanism in cpDNA may resemble that reported for nuclear genomes by Narayanan et al. [33].

According to our hypothesis, DSBs within IRs must have been frequent during angiosperm evolution. However, only those which led to successful IR expansions, and have subsequently been retained in the extant taxa, are detectable. Based on our observations, it is evident that the type of IR-LSC junction appears to be informative, at least at the level of order, and is therefore useful for inferring phylogenetic relationships at this rank and above.

### Expansion of monocot IRs is correlated with the divergence pattern of monocot phylogeny

As shown in Figure 1B, IR-LSC junctions of basal monocots including Acorales, Pandanales and Liliales are usually located at position d. This type might represent a primitive state. In contrast, IR-LSC junctions of the derived monocots, such as Asparagales and Poales, have generally expanded to position f or g. This trend in IR expansion seems to correlate well with the divergence pattern of monocot lineages in the multigene tree of Soltis et al. [27,34], which shows Acorales to be a sister group to other monocots. This correlation connotes the ancient status of the order and the continuous IR expansion experienced by the more terminal and derived lineages, *viz*. Asparagales, Commelinales, Zingiberales, Arecales, Dasypogonaceae and Poales.

It is worth mentioning that in some monocots (e.g. Pandanales and Liliales) the IR-LSC junctions are located at position d, with a truncated *rps19 *gene at IR_A_. According to hypothesis A (Fig. 4), duplication of *rps19 *at IR_A _was due to a second DSB event in IR_A _(Fig. 4, red arrowhead at step 2), followed by a sequence repair supposed to have been terminated within or downstream of the *rps19 *gene. Duplication of the *rps19 *gene will lead to a shift of the IR-LSC junction to position d or f (Fig. 1B). However, in Pandanales and Liliales, the *rps19 *sequences of IR_A _are incomplete or degraded. We considered these common degradations likely to be secondary rather than primary, since the majority of monocot orders have the *trnH-rps19 *clusters (Fig. 1B). Moreover, among the major monocot orders (except Alismatales) the intergenic spacer sequences within the *trnH-rps19 *cluster (Fig. 5B) have a high degree of similarity, suggesting that among the sampled monocots a common DSB event might have taken place adjacent to the *trnH *gene. Therefore, the IRs in Acorales, Pandanales and Liliales are likely to have contracted, causing a shift of the IR-LSC junctions from around position f to position d.

A comparison of the downstream non-coding or spacer sequences of the *rps19 *genes in monocots reveals that the sequences do not have a common origin (Fig. 2B), as they are highly variable and a reliable sequence alignment is impossible except between closely related con-ordinal taxa (e.g. Zingiberales and Asparagales). This indicates that these spacer sequences had diverse origins and are likely to have resulted from independent DSB events occurring at different points within the IRs.

In contrast, it appears that expansion of IR-LSC junctions did not parallel the evolutionary diversification of basal angiosperms and eudicot lineages (Fig. 1A). In type I (Fig. 1), IR expansion downstream of *rps19 *is extremely rare in eudicots, with the exception of Adzuki bean (Perry et al. [18]) and a *Pelargonium *species (Palmer et al. [16], Chumley et al. [11]). According to our hypothesis A (Fig. 4), the scenario of IR expansion in these two eudicots may have different origins from those of monocots and other eudicots (i.e. type II, Fig. 1), with IRs that have expanded downstream of *rps19 *genes. Similarly, significant IR contractions in the basal angiosperm *Illicium oligandrum *(about 1 kb), coriander (4 kb) [13,14], and *Cuscuta reflexa *(about 700 bp to 8 kb) [35] seem to be separate events in their respective lineages.

### Implications of sequences flanking IR-LSC junctions for angiosperm phylogeny

In extant angiosperms, the relationships among the remaining 5 lineages (magnoliids, monocots, eudicots, Chloranthaceae and *Ceratophyllum*) are unresolved [19,26,27]. To what extent the dicot lineage is a sister group of monocots remains uncertain, probably a reflection of the rapid radiation and extinction of early angiosperms soon after they originated [36,37].

Recent phylogenetic analyses based on plastid sequence data have suggested that monocots and eudicots are sister taxa (Graham et al. [38] and Cai et al. [39]), but with low bootstrap support (67% and 72%, respectively). In addition, several lines of evidence have indicated that Ceratophyllaceae could be the sister group of monocots [40-44].

Here we present an alternative view on this issue. As illustrated in Figure 1, an intact *trnH *is duplicated in IR_B _of all monocots, one basal angiosperm (*Nuphar advena*, position c'), and two winteraceous magnoliid species (*Zygogynum paucifolum *and *Drimys granadensis*, position d) [29]. Sequence comparison revealed that only Winteraceae and monocots have highly similar spacer sequences between the *rpl2 *and *trnH *genes (Fig. 5B), suggesting that duplication of *trnH *gene in IR_B _of the two taxa might be common or similar (viz. convergent). On the other hand, Acorales (the most basal lineage in monocots, [27]) has its IR endpoint at position d, suggesting that those lineages with IR-LSC junctions at position b and c' (most Alismatales and Dioscoreales) might have resulted from separate, independent contractions. Our alternative view on the relationships among monocots and their relatives is preliminary, as it is only based on comparison of genic organizations at IR-LSC junctions. Additional molecular and morphological data are required to improve our understanding of monocot phylogeny.

### The presence of two anti-sense strands of *trnH *in monocot IRs is mysterious

The presence of a *trnH-rps19 *cluster in the IRs appears to be a common feature in monocots other than some Alismatids (additional file 1, Fig. 1), in which IR-LSC junctions are located at position b and strongly resemble those of most non-monocot angiosperms. However, alignment of the intergenic spacers between *rpl2 *and *trnH *in some Alismatales (e.g. *Alocasia odora*) and other monocots, basal angiosperms and eudicots (Fig. 5) reveals that sequences of the Alismatids are more similar to other monocots than to non-monocot angiosperms. This implies that IR expansions in some Alismatids might share evolutionary scenarios similar to those proposed for other monocots, and that the short IRs (or IR contraction) in some other Alismatids are likely due to either an early termination of the repair-extension reaction after the first DSB in step 1 of hypothesis A (Fig. 4), or to a contraction after this step.

In monocots, each IR usually contains a *trnH *gene, while in most basal angiosperms and eudicots the gene is rarely present in IR_B _(see Fig. 1A: type I and type II). Why is the duplicated *trnH *gene able to survive in IR_B _of most monocots but is absent, degraded or truncated in most non-monocot angiosperms? In two studied eudicots, *Lotus japonicus *[18] and *Spinacea oleracea *[45], the transcriptional activity of *S10*_*A *_dropped significantly because of either the high transcription levels of the *psbA *and *trnH *genes or the termination of *S10*_*A *_proximal to J_LA _[32]. Therefore, in non-monocot angiosperms, *trnH*-encoded mRNA molecules constitute only one sense strand, transcribed solely by the *psbA *operon rather than by the *S10*_*A *_operon. Because anti-sense RNA molecules may interfere with the normal function of the sense RNA molecules [32], in monocots the mechanism by which anti-sense *trnH *is regulated by two *S10*_*A *_promoters is mysterious. Further study on the evolution and survival of the duplicated *trnH *gene in IR_B _of monocots is desirable.

## Conclusion

Extensive comparisons of the genic organizations flanking the IR-LSC junctions in 123 diversified angiosperm lineages revealed that monocots and non-monocot angiosperms generally have different IR-LSC junction types. Notably, IRs expanded more progressively in monocots than in non-monocot angiosperms, with more LSC genes being converted into IRs. With the exceptions of Alismatales and a few Acorales, the monocot IR_A _regions either encompass a *trnH-rps19 *cluster or extend as far as the 5*' *portion of the *rpl22 *gene, which is typically situated at the LSC region in non-monocot angiosperms. Various expansions of IRs in monocots have resulted in corresponding fluxes of IR-LSC junctions. Our results further indicate that the IR expansions in angiosperms can be explained by initiation of a DSB event and ending at a polyA tract region.

We proposed two hypotheses to explain the evolutionary derivation of the *trnH-rps19 *cluster in the IRs of monocots from an *Amborella-*like ancestor (Fig. 4). Hypothesis A proposes that a DSB event occurs first within the IR_B _of an *Amborella*-like ancestor, and then the free 3*' *end of the broken strand is repaired against the homologous sequence in IR_A_. The repaired sequence extends and results in the duplication of a *trnH *gene in the newly repaired IR_B_. A subsequent DSB event may occur in IR_A _so that the *rps19 *at IR_A _is duplicated, whereby a *trnH-rps19 *cluster is created. Hypothesis B assumes that *rps19 *is duplicated or converted before the duplication of *trnH *via a DSB event that occurs at IR_A_.

It is worth noting that IR expansions in monocots appear to correlate well with the divergence pattern of monocot phylogeny. The present study highlights the use of sequences flanking the IR-LSC junctions to address the evolutionary dynamics of IRs from basal angiosperms to monocots. Taken together with the evidence from the IR-LSC junctions, we conclude that (i) monocots may be closely related to the Winteraceae (magnoliids) than to other basal angiosperms or eudicots, (ii) the shorter IRs in Alismatids are probably due to either an early termination of repair-extension after the first DSB, or to a contraction after this step, and (iii) the duplicated *trnH *genes in the IR_B _of most monocots and non-monocot angiosperms have distinct fates, which are likely regulated by different expression levels of *S10*_*A *_and *S10*_*B *_promoters. Further study is needed to unravel the evolutionary significance or advantage of the presence of an additional *trnH *in monocot IRs, and of IR expansion in more recently diverged monocots.

## Methods

### Plant materials and DNA preparation

Species sampled in this study were listed in the additional file 1. Total cellular DNA was extracted using the method of Saghai-Maroof et al. [46]. The extracted DNAs were used directly for PCR amplification.

### PCR amplification

Primer design was based on published sequence data for conserved regions flanking the IR-LSC junctions. The J_LA _regions were amplified with the primer pair *rpl2-psbA-*F3 and *rpl2-psbA-*R2, which correspond to the 3*' *end of *rpl2 *and the 5*' *end of *psbA *respectively (Fig. 1). The J_LB _region was amplified using two forward primers, *rps3*-F1 and *rps3*-F2, that respectively pair with a reverse primer *rps3*-*rpl2*-R2. The sequences of these primers are listed in Table 1. Amplicons were cleaned using the Gel Extraction System (Viogene, Taipei) and cloned into a pGEM T-Easy vector (Promega, Fitchsburg). Plasmid DNAs were purified using the Plasmid DNA Miniprep System (Viogene) and sequenced on an ABI 3730 automated sequencer (Applied Biosystems, Foster City). For each species two independent PCR clones were sequenced. Sequence alignments were made using GeneDoc (Ver. 2.6.02.)

**Table 1:** Primers used for analyses of IR-LSC junctions and in RT-PCR

Primer number	Name	Sequence	Application
1	*rpl2*-*psbA*-F1	5'-GACCCTAATCGAAATGCRTMCATTTG-3'	IR_A_
2	*rpl2*-*psbA*-F2	5'-TAATTGGAGATACYATTKKTTCTGGTACA-3'	IR_A_
3	*rpl2*-*psbA*-R1	5'-ATGGCDTTCAAYYTRAAYGGMTTYAATTT-3'	IR_A_
4	*rpl2*-*psbA*-R2	5'-CTTGGTATGGARGTMATGCAYGARCGTAA-3'	IR_A_
5	*rps3*-*rpl2*-F1	5'-GYTAAYTCRATRRCYTTTTTCATTGC-3'	IR_B_
6	*rps3*-*rpl2*-F2	5'-AWABYYYKTTGGTTKTGMRAACCA AA-3'	IR_B_
7	*rps3*-*rpl2*-R1	5'-AATGGGAAATGCCCTACCTTTG-3'	IR_B_
8	*rps3*-*rpl2*-R2	5'-GTAGTAAGAGGRGTRGTTATGAACCC-3'	IR_B_
9	*rpl22*-F1	5'-TRRTTTATTCBGCAGCVGCAAATGC-3'	IR_B_
10	*rps3*-F1	5'-ATAWATTCYGCAAGAATRTTAGG-3'	IR_B_
11	*rps3*-F2	5'-AGTCKGAAACCRAGTGGATTT-3'	IR_B_
12	*rpl2*-*psbA*-F3	5'-GGTAARCGYCCYGTAGTAAGAGG-3'	IR_A_
13	*trnH*-*psbA*-F1	5'-GGCGAACGACGGGAATTGAAC-3'	IR_A_
14	*trnH*-rev	5'-GGATGTAGCCAAGTGGATCAAGG-3'	IR_A_

### Reverse Transcriptase-Polymerase Chain Reaction (RT-PCR) Assay

To verify the transcription of *trnH*-*rps19 *that flanks the IR_A _region, total RNAs were extracted and purified by RNeasy^® ^Plant Mini Kit (Qiagen, Hilden). The resulting RNAs were reversely transcribed to synthesize cDNA with Superscript II reverse transcriptase (Invitrogen, Indianapolis) and a specific primer (either *trnH-psbA*-F1 or *trnH*-rev), according to the manufacturer's protocol. The two synthesized cDNAs were then used with the primer pair *trnH-psbA*-F1 and *rpl2-psbA*-R2 to amplify a 674 bp fragment, and the primer pair *trnH*-rev and *rpl2-psbA*-F3 to amplify a 298 bp fragment. Each of the two reactions was conducted under the following conditions: 94°C for 5 min, followed by 30 cycles of 94°C for 30s, 55°C for 30s, and 72°C for 30s, and ending with an extension of 72°C for 10 min.

## Abbreviations

cpDNA, chloroplast genome; IR, inverted repeat; SSC, small single copy; LSC, large single copy; bp, base pair; J_LA_, junction between LSC and IR_A_; J_LB_, junction between LSC and IR_B_; DSB, double-strand break; RT-PCR: reverse transcriptase-polymerase chain reaction.

## Authors' contributions

SMC conceived the study. CLC, CLW, TMS and RJW carried out the sequence analysis, and CCC provided the unpublished orchid data. CLC and CLW prepared the sequence data and submitted it to GenBank. CLC prepared the figures. RJW, SMC, and CLC wrote the manuscript. All authors read and approved the final manuscript.

## Supplementary Material

Additional file 1 Studied taxa and their GenBank accession numbers, references and IR-LSC junction positions. This table (Table S1) provides detailed information about the studied 123 taxa, including 12 basal angiosperms, 16 magnoliids, 62 eudicots, and 33 monocots, involved in the analysis. Click here for file
