# Supplementary material for: Dynamics and evolution of the inverted repeat-large single copy junctions in the chloroplast genomes of monocots
Source: BMC Evol Biol. 2008 Jan 31;8:36. doi: 10.1186/1471-2148-8-36 (PMC2275221; doi:10.1186/1471-2148-8-36)
Supplement: Additional file 1 — Studied taxa and their GenBank accession numbers, references and IR-LSC junction positions. This table (Table S1) provides detailed information about the studied 123 taxa, including 12 basal angiosperms, 16 magnoliids, 62 eudicots, and 33 monocots, involved in the analysis. [file 1471-2148-8-36-S1.PDF]

Table S1: Studied taxa and their GenBank accession numbers, references and IR-LSC junction positions.

Taxon	GenBank Accession Number	Reference	IR-LSC Junction Position
**Basal Angiosperms**			
Amborellaceae			
Amborella trichopoda	NC_005086	Goremykin et al. 2003 [29]	b
Nymphaeaceae			
Brasenia schreberi	AB331296, AB331347	Current study	b
Euryale ferox	AB331295, AB331346	Current study	b
Nymphaea alba	NC_006050	Goremykin et al. 2004 [47]	b
Nuphar advena	NC_008788	Raubeson et al. 2007 [48]	c'
Austrobaileyales			
Kadsura japonica	AB331304, AB331328	Current study	a
Schisandra arisanensis	AB331282, AB331329	Current study	e
Ceratophyllales			
Ceratophyllum demersum	AB331297, AB331348	Current study	b
	EF614270	Moore et al. 2007 [49]	
Chloranthaceae			
Chloranthus oldhami	AB331284, AB331333	Current study	c
Chloranthus spicatus	NC_009598	Hansen et al. 2007 [13]	c
Sarcandra glabra	AB331291, AB331341	Current study	c
**Magnoliids**			
Canellales			
Canella winterana	AB331300, AB331352	Current study	c
Zygogynum pauciflorum	AB331299, AB331351	Current study	d
Drimys granadensis	DQ887676	Cai et al. 2006 [39]	d
Piperales			
Piper coenoclatum	DQ887677	Cai et al 2006 [39]	b
Piper kadsura	AB331285, AB331334	Current study	b
Houtuynia cordata	AB331281, AB331326	Current study	b
Saururus chinensis	AB331280, AB331325	Current study	b
Laurales			
Calycanthus fertilis	AJ428413	Goremykin, 2003 [29]	a'
Calycanthus floridus	NC_004993	Goremykin, 2003 [30]	a'
Cinnamomum camphora	AB331294, AB331344	Current study	b
Litsea hypophaea	AB331293, AB331343	Current study	b
Magnoliales			
Artabotrys hongkongensis	AB331301, AB331363	Current study	b

Fissistigma glaucescens	AB331302, AB331364	Current study	b
Michelia compressa	AB331279, AB331321	Current study	b
Liriodendron chinense	AB331303, AB331360	Current study	b
Liriodendron tulipifera	NC_008326	Cai et al. 2006 [39]	b
**Eudicots**			
Ranunculales			
Nandia domestica	DQ923117	Moore et al. 2006 [50]	e
Ranunculus japonica	AB244028, AB331322	Current study	c
Ranunculus macranthus	NC_008796	Raubeson et al. 2007 [48]	c
Stauntonia hexaphylla	AB331289, AB331339	Current study	e
Proteales			
Grevillea robusta	AB331286, AB331335	Current study	b
Nelumbo nucifera	AB331309, AB331327	Current study	b
Platanus occidentalis	DQ923116	Moore et al. 2006 [50]	e
Sabiaceae			
Meliosma rigida	AB331288, AB331338	Current study	b
Trochodendraceae			
Trochodendron aralioides	AB331290, AB331340	Current study	b
Buxaceae			
Buxus microphylla	AB331298, AB331349	Current study	b
	NC_009599	Hansen et al. 2007 [13]	
Caryophyllales			
Spinacea oleracea	NC_002202	Schmitz-Linneweber et al. 2001 [51]	e
Dipsacales			
Viburnum awabuki	AB331287, AB331337	Current study	b
Asterales			
Helianthus annuus 'HA383'	DQ383815	Timme et al. unpublished	e
Lactuca sativa	AP007232	Kanamoto et al. unpublished	b
	DQ383816	Timme et al. unpublished	
Apiales			
Panax schinseng	NC_006290	Kim and Lee 2004 [52]	e
Tetrapanax papyriferus	AB331283, AB331331	Current study	e
Daucus carota	DQ898156	Ruhlman et al. 2006 [53]	e
Gentianales			
Coffea arabica	NC_008535	Samson et al. 2007 [54]	e
Lamiales			
Digitalis purpurea	Z71251, Z71252	Goulding et al. 1996 [15]	e
Epifagus virginiana	M81884	Wolfe et al. 1992 [55]	e

Jasminum nudiflorum	DQ673255	Lee et al. 2007 [56]	b
Solanales			
Atropa belladonna	NC_004561	Schmitz-Linneweber et al. 2002 [57]	b
Nicotiana acuminata	Z71253, Z71254	Goulding et al. 1996 [15]	e
Nicotiana alata	Z71239	Goulding et al. 1996 [15]	e
Nicotiana attenuata	Z71242	Goulding et al. 1996 [15]	e
Nicotiana bigelovii	Z71225, Z71226	Goulding et al. 1996 [15]	e
Nicotiana debneyi	X00796, X00798	Zurawski et al. 1984 [45]	e
Nicotiana glauca	Z71244	Goulding et al. 1996 [15]	e
Nicotiana glutinosa	Z71238	Goulding et al. 1996 [15]	e
Nicotiana palmeri	Z71234, Z71235	Goulding et al. 1996 [15]	e
Nicotiana plumbaginifolia	Z71240, Z71241	Goulding et al. 1996 [15]	e
Nicotiana nudicaulis	Z71232	Goulding et al. 1996 [15]	e
Nicotiana sylvestris	Z71233	Goulding et al. 1996 [15]	e
	AB237912	Yukawa et al. 2006 [58]	
Nicotiana tabacum 'Bright Yellow 4'	NC_001879	Shinozaki et al. 1986 [3]	e
Nicotiana tabacum cultivars	Z71227- Z71230, Z71236, Z71237	Goulding et al. 1996 [15]	e
Nicotiana tomentosiformis	AB240139	Yukawa et al. 2006 [58]	e
Nicotiana velutina	Z71243	Goulding et al. 1996 [15]	e
Petunia hybrida	X12856	Aldrich et al. 1988 [59]	e
Solana bulbocastanum	DQ347958	Daniell et al. unpublished	e
Solanum lycopersicum 'LA3023'	DQ347959	Daniell et al. unpublished	e
Solanum lycopersicum 'Moneymaker'	Z71245, Z71246	Goulding et al. 1996 [15]	e
Solanum lycopersicum	AM087200	Kahlau et al. 2006 [60]	e
Solanum nigrum	Z71249, Z71250	Goulding et al. 1996 [15]	e
Solanum tuberosum	Z71247, Z71248	Goulding et al. 1996 [15]	e
Solanum tuberosum 'Desiree'	DQ386163	Gargano et al. unpublished	e
Vitaceae			
Vitis vinifera	NC_007957	Jansen et al. 2006 [31]	a'
Myrtales			
Oenothera elata ssp. *hookeri*	NC_002693	Hupfer et al. 2000 [61]	e
Eucalyptus globulus	NC_008115	Steane et al. 2005 [62]	b
Syzygium jambos	AB331292, AB331342	Current study	e

Brassicales			
Arabidopsis thaliana	NC_000932	Sato et al. 1999 [63]	b
Sinapis alba	X17331	Nickelsen and Link 1990 [64]	e
Sapindales			
Citrus sinensis	NC_008334	Bausher et al. 2006 [65]	e'
Malvales			
Gossypium barbadense	AP009123	Ibrahim et al. 2006 [66]	b
Gossypium hirsutum	NC_007944	Lee et al. 2006 [67]	b
Malpighiales			
Populus alba	NC_008235	Okumura et al. unpublished	e'
Fabales			
Glycine max	X06429	Spielmann et al. 1988 [68]	e
	NC_007942	Saski et al. 2005 [69]	
Lotus japonicus	NC_002694	Kato et al. 2000 [70]	b
Vigna angularis	AF536225, AF536226	Perry et al. 2002 [18]	e'
Cucurbitales			
Cucumis sativus	NC_007144	Plader et al. unpublished	b
Rosales			
Elaeagnus formosana	AB244029, AB331323	Current study	c'
Morus indica	DQ226511	Ravi et al. 2006 [71]	b
**Monocots**			
Acorales			
Acorus calamus	AJ879453	Goremykin et al. 2005 [72]	d
	AB331256, AB331311	Current study	
Acorus gramineus	AB331257, AB331312	Current study	d
Alismatales			
Alisma canaliculatum	AB331258, AB331313	Current study	d
Caldesia grandis	AB331310, AB331372	Current study	b
Alocasia odora	AB331277, AB331361	Current study	b
Syngonium podophyllum	AB331278, AB331362	Current study	b
Najas indica	AB331276, AB331358	Current study	b
Potamogeton crispus	AB331275, AB331371	Current study	c'
Pandanales			
Pandanus odoratissimus	AB331260, AB331316	Current study	d
Dioscoreales			
Dioscorea elephantipes	NC_009601	Hansen et al. 2007 [13]	c'
Liliales			
Lilium formosamum	AB331270, AB331357	Current study	d
Asparagales			
Asparagus densiflorus	AB331262, AB331318	Current study	f

Crinum asiaticum	AB331268, AB331356	Current study	g
Dendrobium sp.	AB331272, AB331367	Current study	g
Oncidium flexuosum	AB331305, AB331366	Current study	g
Phalaenopsis aphrodite	NC_007499	Chang et al. 2006 [20]	g
Commelinales			
Murdannia keisak	AB331265, AB331370	Current study	g
Zingiberales			
Hedychium coronarium	AB331263, AB331324	Current study	f
Arecales			
Livistona chinensis	AB331261, AB331317	Current study	f
Dasypogonaceae			
Dasypogon bromeliifolius	AB331273, AB331368	Current study	f
Calectasia intermedia	AB331274, AB331369	Current study	f
Poales			
Scirpus ternatanus	AB331264, AB331336	Current study	f
Agrostis stolonifera	EF115543	Saski et al. unpublished	f
Bambusa oldhamii	AB331267, AB331350	Current study	f
Digitaria sanguinalis	AB331271, AB331359	Current study	f
Hordeum vulgare ssp. *vulgare* 'Morex'	EF115541	Saski et al. unpublished	f
Oryza nivara	NC_005973	Masooda et al. 2004 [73]	f
Oryza sativa	NC_001320	Hiratsuka et al. 1989 [74]	f
Saccharum officinarum	NC_006084	Asano et al. 2004 [75]	f
Sorghum bicolor 'BTx623'	EF115542	Saski et al. unpublished	f
Triticum aestivum	NC_002762	Ogihara et al. 2002 [76]	f
Zea mays	NC_001666	Maier et al. 1995 [9]	f
Typha angustata	AB331259, AB331315	Current study	f
